# Probable Drug Interaction Between Etanercept and Cyclosporine Resulting in Clinically Unexpected Low Trough Concentrations: First Case Report

**DOI:** 10.3389/fphar.2020.00939

**Published:** 2020-06-26

**Authors:** Haini Wen, Dongping Chen, Jiaqian Lu, Zheng Jiao, Bin Chen, Bin Zhang, Chaoyang Ye, Li Liu

**Affiliations:** ^1^ Department of Clinical Pharmacy, Pharmacy, Shuguang Hospital Affiliated to Shanghai University of Traditional Chinese Medicine, Shanghai, China; ^2^ Department of Nephrology, TCM Institute of Kidney Disease, Shuguang Hospital Affiliated to Shanghai University of Traditional Chinese Medicine, Shanghai, China; ^3^ Department of Pharmacy, Ruijin Hospital Affiliated to Shanghai Jiaotong University School of Medicine, Shanghai, China; ^4^ Department of Pharmacy, The Chest Hospital Affiliated to Shanghai Jiaotong University School of Medicine, Shanghai, China; ^5^ Department of Clinical Laboratory, Shuguang Hospital Affiliated to Shanghai University of Traditional Chinese Medicine, Shanghai, China

**Keywords:** biological therapy, cyclosporine, etanercept, trough level, drug-cytokine interaction, inflammatory disease, case report

## Abstract

Preclinical studies have shown that anti-cytokine therapies could alter drug dispositions through affecting cytochrome P450 synthesis; however, evidence and case reports evaluating clinical relevance of this interaction are scarce. This is the first reported case of interaction between cyclosporine (CsA) and etanercept in a 42-year-old male patient with ankylosing spondylitis and immunoglobulin A nephropathy in whom cytokine levels were monitored both before and after CsA initiation. The initiation of etanercept led to at least 2.5-folds increase in total clearance of CsA. After comprehensive assessment and stepwise exclusion of alternative causes, it was considered that inflammation resolution with etanercept administration has highly induced clearance of CsA, probably mediated by interleukin-2. The case has shown that co-administration of CsA and anti-cytokine therapies such as etanercept needs close monitoring of trough levels. Physicians and pharmacists should be aware of similar interactions especially when the biological therapy is initiated or discontinued and for patients undergoing acute inflammation phase. Monitoring cytokine levels should be considered when drug-cytokine interaction is suspected.

## Introduction

Cyclosporine (CsA) is a calcineurin inhibitor commonly used to prevent post-transplant rejections or to treat immune-mediated diseases. Whole-blood CsA levels are routinely monitored to achieve therapeutic range as well as to avoid toxicities. CsA levels can be altered by many concurrent medications considering that CsA is a substrate of cytochrome P450(CYP) 3A4, CYP3A5, and P-glycoprotein (Watkins, 1990). Etanercept is a tumor necrosis factor (TNF) blocking agent with proven efficacy and safety in the treatment of autoimmune diseases such as ankylosing spondylitis (Murdaca et al., 2018). It is a fusion protein consists of two p75 TNF receptors bound to the fragment crystallizable region of immunoglobulin G. No specific drug interaction studies were conducted with etanercept during its clinical development, as it was presumed that etanercept would be metabolized as peptides and amino acids rather than as a CYP450 substrate (Zhou, 2005). However, later studies showed that anti-cytokine therapies can alter drug dispositions through “drug-cytokine interaction” (Goralski et al., 2018).

Cytokines, such as interleukins (IL), interferons, and TNFs are hormone-like glycoproteins that regulate human immunologic responses as mediators (Goralski et al., 2018). The term “drug-cytokine interaction” is used to describe how cytokines interact with drug-metabolizing enzymes or drug transporters and therefore influence drug dispositions. Though first proposed back in 1966 and was well supported by animal studies since then, drug-cytokine interaction is barely considered relevant in clinical scenarios (Morgan et al., 2008). Moreover, how cytokine therapies such as etanercept would change exposure of concomitant drugs lacks systemic evaluation and clarification (Huang et al., 2010). Here, we describe the first reported case of a probable drug-drug interaction between CsA and etanercept, supported by the mechanism of “drug-cytokine interaction.”

## Case Presentation

A 42-year-old Chinese male patient with past medical history of ankylosing spondylitis (HLA-B27 positive), hypertension, and type 2 diabetes mellitus was admitted to Shuguang Hospital (Shanghai, China) for renal biopsy in June 2018 after having persistent proteinuria and serum creatinine elevation. He was taking metformin and levoamlodipine (active enantiomer of amlodipine) for diabetes and hypertension, respectively; for ankylosing spondylitis, he was treated with celecoxib, sulfasalazine, and methotrexate, but all three medications were discontinued after the onset of proteinuria 4 years ago, and etanercept and losartan were started. On admission, his back pain was well-controlled, with Bath Ankylosing Spondylitis Disease Activity Index (BASDAI) score 3.0, his blood pressure was 120/76 mmHg, and hemoglobin A1C was 7.1% (Zochling, 2011).

**Figure 1:**
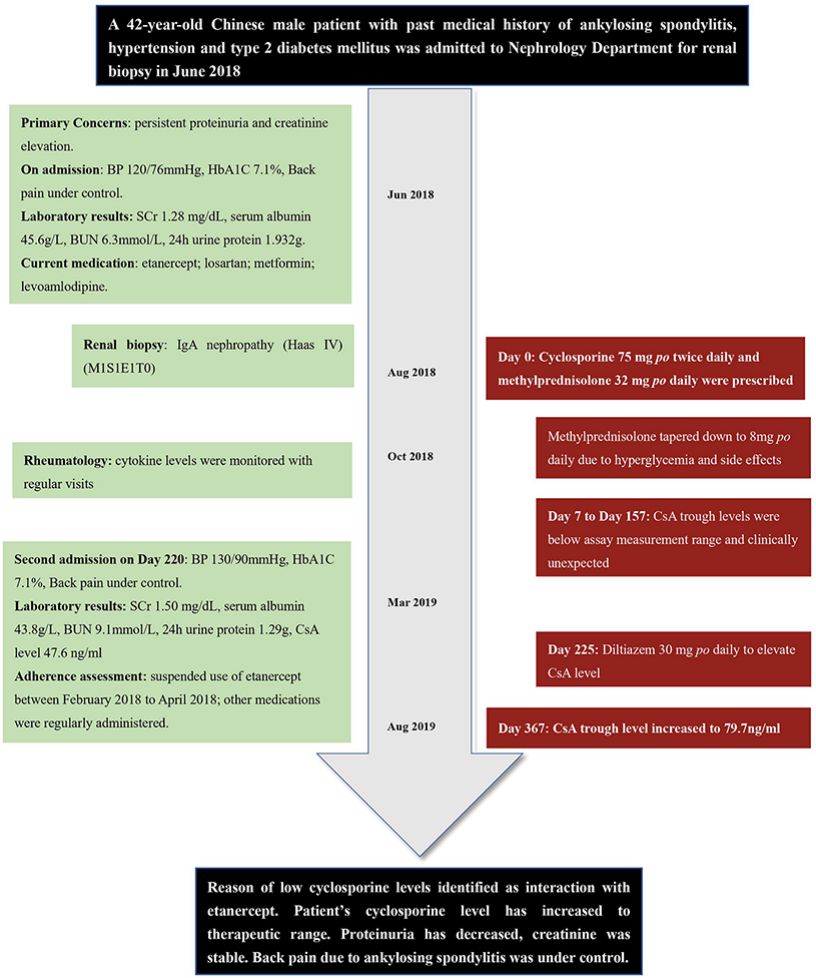
Timeline of the presented case report. BP, blood pressure; SCr, serum creatinine; BUN, blood urea nitrogen; CsA, cyclosporine.

The patient underwent renal biopsy and was diagnosed with IgA nephropathy (Haas IV) (M1S1E1T0) after admission. Microemulsion formulated CsA 75 mg (2 mg/kg/d, patient weight 75 kg) twice daily and methylprednisolone 32 mg (0.4 mg/kg/d) once daily orally were prescribed for IgA nephropathy treatment starting August 2018. For this case report, the day of first CsA administration was set as day 0. Due to occurrence of hyperglycemia and intolerance of gastrointestinal side effects, methylprednisolone was then slowly tapered down to 8 mg once daily within 90 d. Seven and 17 d after CsA initiation, patient's CsA trough concentrations (C0, day 7 and day 17) were both < 30 ng/ml. Accordingly, the physician assessed patient compliance and emphasized on the importance of taking CsA on time (9 am and 9 pm) and sampling C0 on time (between 8:30 am and 9:00 am). On day 45 and day 157, patient's CsA trough concentration was < 30 and 39.8 ng/ml, respectively. Of note, patient's serum creatinine and albumin were relatively stable during this period of time. Liver function was normal. Dosage of CsA stayed the same according to physician's clinical judgment. As patient also visited his rheumatologist in this hospital, his cytokine levels were available both before and after CsA initiation. Timeline of the presented case is shown in **Figure 1**.

According to hospital laboratory, the analytical method employed by hospital laboratory was chemiluminescent magnetic microparticle immunoassay (CMIA) with ARCHITECT CsA kit (Abbott Laboratories), with a measurement range of 30–1,500 ng/ml. Therefore, the physician doubted the accuracy of the assay results. Referring to the laboratory sampling records, all patient's blood samples were correctly collected between 8:00 am to 9:00 am. Moreover, calibration and quality control procedures for the assay were done every 24 h according to guideline for quantitative analysis of biological samples by Chinese Pharmacopoeia (2015). After discussion, the team proposed several possible explanations for the unexpectedly low CsA level: 1. The values were correct and reflected non-compliance of CsA administration; 2. The values were correct and reflected possible factors increased CsA metabolism, such as physical condition changes or drug-drug interactions; 3. The values were artifactual and reflected assay interference, such as by heterophilic antibodies.

## Investigations and Results

The patient was admitted again on day 220. To find out reasons of extremely low CsA levels, pharmacist conducted medication reconciliation and evaluated medication adherence for the patient. Patient's adherence was assessed by checking patient's refill records, as well as self-reported by patient; adherence result was recorded as percentage of doses taken from total doses prescribed (Hess et al., 2006). Patient's adherence of current medications was as following: CsA 75 mg twice daily (9:00 am and 9:00 pm, for 220 d, 100% adherence); methylprednisolone 8 mg once daily (for 220 d, 100% adherence); metformin 500 mg once daily (for 3 years, > 95% adherence), levoamlodipine 2.5 mg once daily (for 5 years, > 95% adherence), losartan 50 mg once daily (for 4 years, > 95% adherence); etanercept 25 mg subcutaneously every 10 d (for 4 years, 70% adherence) as maintenance dosing regimen. No food interaction (including alcohol) observed considering patient's dietary habits. No pre-existing gastrointestinal conditions. Patient had irregular refill records of etanercept between April 2017 to April 2018. According to the patient, his very recent suspension of etanercept use was between February 2018 to April 2018 (day −188 to day –126) due to frequent business traveling, which were before CsA initiation. As interruption of etanercept had caused frequent eruptions of back pain, the patient changed his job to avoid frequent traveling and resumed regular etanercept use since then. In general, patient showed his strong willingness to manage his health condition responsibly. Also, he had been taking CsA on time and sampling as directed since first administration.

To validate laboratory reports and exclude possibilities of assay interference, we delivered two whole blood samples drawn at the same time (C0, 8:50 am before medication administration, day 157) to hospital laboratory as usual and pharmacy laboratory for liquid chromatography-mass spectrometer (LC/MS/MS) CsA determination simultaneously.

LC/MS/MS was performed on UFLC Chromatographic System and API4000 Qtrap Mass Spectrograph. Methodology of LC/MS/MS determination of CsA was systemically validated before sample determination. Standard calibration curves were constructed when method's accuracy, selectivity, precision, recovery rate met requirements of the guideline for quantitative analysis of biological samples by Chinese Pharmacopoeia (2015). Detailed validation and sample determination process of LC/MS/MS are provided in Supplementary Material. As a result, patient's sample concentration was 59.4 ng/ml according to LC/MS/MS method. Meanwhile, the result provided by hospital laboratory by CMIA method was 47.6 ng/ml. As two results were comparable to each other, previous speculation of assay interference caused by heterophilic antibodies was excluded.

**Figure 2:**
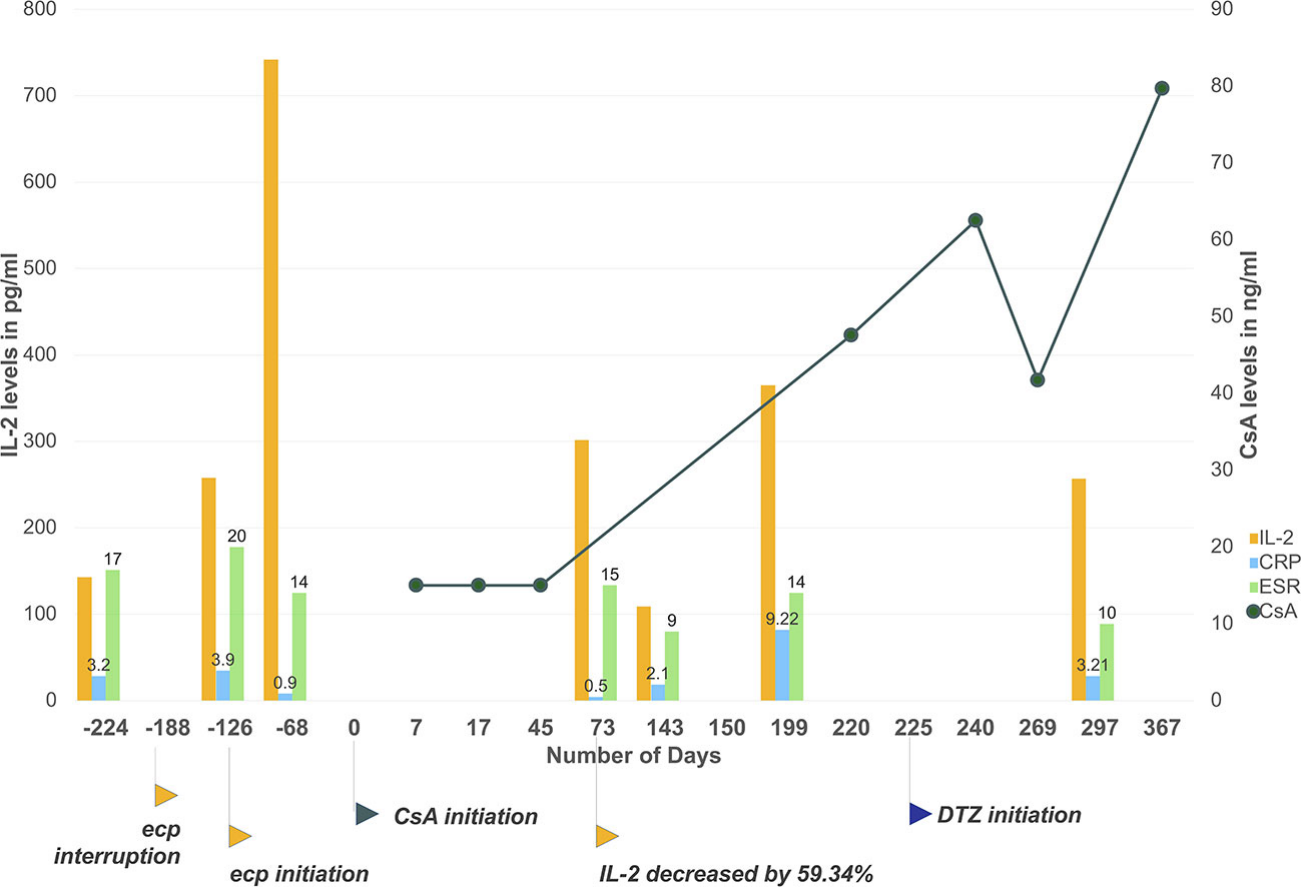
Patient's CsA level, IL-2, and ESR/CRP levels monitoring during the time. Etanercept interruption has led to cytokine level fluctuations and clinically unexpected CsA trough levels. The initiation of etanercept has induced a steep decline of IL-2, ESR and CRP levels due to inflammation resolution. Initiation of CsA overlapped with the decline. As a result, the first three CsA level results were lower than the measurement range of applied immunoassay. From day 225 to day 367, CsA trough levels slowly increased to 79.7 ng/ml with co-administration of diltiazem and under more stable cytokine levels. Day 0 set as the first day of CsA administration. Plotted CsA level results were measured with chemiluminescent magnetic microparticle immunoassay by hospital laboratory. Results below measurement range were taken as 15 ng/ml (half of assay lower detection limit). Csa, cyclosporine; ecp, etanercept; ESR, erythrocyte sedimentation rate; CRP, Creactive protein. Applied unit for CRP is mg/ml, for ESR is mm/h.

Meanwhile, we noticed correlation between suspension of etanercept use and irregularity of CsA trough levels, probably mediated by IL-2. As shown in **Figure 2**, patient's discontinuation of etanercept from February to April 2018 (day −188 to day −126) has led to 422.5% increase of IL-2 before first dose of CsA, during which patient experienced intolerable breakthrough back pain (BASDAI score 5.7 at day −126). Re-initiation of etanercept led to declined disease activity. As shown in **Figure 2**, patient's inflammatory indicators of erythrocyte sedimentation rate (ESR) and C-reactive protein (CRP) continuously decreased after etanercept use, also patient reported significant pain relief (BASDAI score 2.8 on day 73). Of noted, patient's IL-2 level dropped by 59.34% on day 73. Initiation of CsA administration overlapped with the time of patient's inflammation remission. As a result, the first three CsA trough levels of day 7, 17, and 45 were extremely low and undetectable. Therefore, we suspected interaction between CsA and etanercept in this particular patient, whose initiation of etanercept therapy seemed to be correlated to high clearance of CsA, leading to decreased cytokine activities that would induce higher CYP synthesis and faster metabolism of CYP substrates.

According to the Drug Interaction Probability Scale (DIPS), our case is classified as a probable drug-drug interaction, with a score of 6 (Horn et al., 2007). To quantify the extent of drug interaction, it is presented that a 59.34% drop of IL-2 level for 141 d due to etanercept initiation has depressed CsA trough level by at least 60% for 45 d at the lowest estimation, assuming patient should have reached 75 ng/ml with current CsA dosage and taking first three results as 30 ng/ml. According to the equation C_ss_ = (dose × bioavailability)/(dosing interval × Cl_total_) and based on the assumption above, etanercept initiation has led to at least 2.5-folds increase in total clearance of CsA.

In order to achieve therapeutic concentration of CsA for IgA nephropathy treatment, patient was prescribed with diltiazem 30 mg once daily orally at discharge from day 225 to inhibit CYP3A4 metabolism. Dosage of CsA remained unchanged according to physician's clinical judgment. From day 225 to day 367, CsA trough level slowly increased to about 79.7 ng/ml, which also corresponded to more stable cytokine levels. Detailed laboratory results of cytokine levels, ESR, and CRP values are provided in **Supplementary Table 1**.

## Discussion

Major drug references such as Lexicomp^®^ and Micromedex^®^ showed no significant drug interactions between patient's co-medications. The team noticed that the package insert of adalimumab (Humira) suggested that drug concentration of CsA should be monitored during initiation and discontinuation of adalimumab therapy, as adalimumab initiation could increase CYP synthesis and lead to decreased CsA concentrations (AbbVie Inc, 2020). An English-language literature search (PubMed, Web of Science; database inception to February 2020) identified no case report of drug interaction between etanercept and CsA, nor between adalimumab and CsA. This is the first case of drug interaction between etanercept and CsA with quantification techniques and suggested mechanism supported by patient's inflammatory indicators.

While therapeutic proteins are increasingly approved and prescribed over the world, our understanding of drug interactions associated with them is still premature and sometimes controversial. Preclinical studies revealed that exposure to cytokines such as IL-1, IL-2, IL-6, and TNF-alpha can reduce hepatic cytochrome P450 metabolism through transcriptional regulation of CYP synthesis (Goralski et al., 2018). In patients receiving biologicals, the resolution of inflammation by anti-cytokine therapies can therefore increase drug clearance, due to reversal of the down-regulation and induction of the suppressed enzymes (Morgan, 2009). However, clinical studies are not always drawing consistent conclusions. While many clinical studies on therapeutic protein-drug interactions showed negative or insignificant results, some researchers pointed out that most trials were poorly designed without considering the underlying mechanisms or/and disease states of patients (Lee et al., 2010; Zhou and Sharma, 2016; Jing et al., 2020). Admittedly, drug-cytokine interaction is complicated in real clinical scenarios as it involves disease states, inflammation characteristics and severity, as well as related drugs. As of now, it appears that the degree of inflammation positively correlates to interaction potential; therefore significant drug-cytokine interactions are mostly observed during acute inflammation with immune response or induction/discontinuation of cytokine-therapies (Zhou and Sharma, 2016). By monitoring both cytokine and affected drug levels, our case demonstrated drug-cytokine interaction was of significant clinical importance—cytokines and anti-cytokine biologicals can affect therapeutic effects or toxicities of co-medications, which required close monitoring during clinical practice, especially for patients with infection, inflammation or cancer (Morgan et al., 2008; Veringa et al., 2017; Goralski et al., 2018).

Previous studies found that etanercept would not affect pharmacokinetics of digoxin and warfarin in healthy subjects (Zhou, 2005). However, our case revealed possible interaction between etanercept and CsA in a patient with ankylosing spondylitis and IgA nephropathy, with the interaction correlated with changes in IL-2 levels. It should also be noted that the described interaction between CsA and etanercept would mostly happen with discontinuation or induction of etanercept, which may explain the reason that there were zero report of similar cases in the past. Strehlau et al. (2000) found basiliximab altered CsA metabolism in pediatric transplant recipients, which shared common features with our case such that both changes of CsA trough levels seemed to be IL-2 mediated. CsA is a major substrate of CYP3A4 and minor substrates of p-glycoprotein. *In vitro* studies have shown that IL-2 regulates expression of CYP3A, and both IL-2 and TNF-alpha regulate expression of p-glycoprotein (Elkahwaji et al., 1999; Zídek et al., 2009). Similar to our presented case, one clinical trial (NCT02017639) has shown that induction of sarilumab resulted in a reduction in exposure of simvastatin by 45%, when simvastatin was given 7 d after single-dose sarilumab, due to reversal of IL-6-mediated CYP3A4 suppression in patients of active rheumatoid arthritis (Lee et al., 2017).

Interestingly, while etanercept showed good clinical response in our patient, as indicated by decline of BASDAI score and inflammatory indicators such as ESR and CRP, cytokine levels such as IL-2 and TNF-alpha increased first for 8 weeks before they further decreased within normal ranges (Murdaca et al., 2018). The phenomenon, suggesting that elevated cytokine levels did not always correspond with increased disease activity, was also observed in other clinical studies involving patients treated with etanercept under conditions of rheumatic autoimmune diseases (Schulz et al., 2014; Takeshita et al., 2015; Walters et al., 2016). For example, Walters et al. (2016) reported that TNF-alpha and IL-17 increased significantly for about 4 and 8 weeks respectively in etanercept but not adalimumab-treated subjects, while the clinical improvement of both treatments was similar. Besides proposed explanations such as counter regulatory effects on T cells or serum TNF-alpha stabilizing effects of etanercept, the mechanism associated with the phenomenon needs further research (Zou et al., 2003; Nowlan et al., 2005). Combined with major findings in the presented case, the mentioned phenomenon might also imply a higher possibility of “drug-cytokine interaction” for etanercept therapies.

Several limitations of this patient case report should be addressed. First, DIPS was employed to rate likelihood of the presented case. Though DIPS is considered as the most appropriate and the only published method to evaluate individual case reports, it is likely to yield low causation scores when information about similar drug interactions is limited, such as the presented case (Agbabiaka et al., 2008; Scheife et al., 2015). Second, the cytokine levels of this patient were not drawn simultaneously with CsA levels. Around initiation of CsA therapy, cytokine levels were available for 68 d before initiation and 73 d after (**Figure 2**, **Supplementary Table 1**). The trend was described by assuming that cytokine levels were decreasing in general due to etanercept initiation. Third, the dosage of CsA remained the same in the case. For IgA nephropathy treatment, no agreed target range was recommended by clinical guidelines (Obrișcă et al., 2019). Empirically, the dosage of CsA for IgA nephropathy treatment ranges between 100–5 mg/kg/d per different clinical practice, and trough levels of 70–180 ng/ml are achieved for most patients (Liu et al., 2014; Song et al., 2017). For this particular patient, the team chose to observe and monitor disease activity closely instead of increasing dosage instantly to avoid potential adverse events due to immunosuppressive effects, as well as considering protecting the patient's renal function. However, for clinical scenarios with more strict target concentration ranges, more aggressive intervention would be employed. Forth, while the method of medication adherence assessment employed in the investigation was refill records checking combined with patient's self-report to increase reliability, it is still imperfect and might fail to identify patient's non-adherence to CsA with a small possibility.

During investigation of this patient case, assay interference from endogenous proteins or peptides was also considered as a possible cause of clinically unexpected CsA concentrations, as we noticed that false results due to immunoassay interference were common during therapeutic drug monitoring (Tsoi et al., 2019). For example, it was reported in literature that patients receiving etanercept may develop antibodies that could interfere with immunoassays, which posed one challenge for reading laboratory results of patients taking biological therapeutics (Russell et al., 2000; Bolstad et al., 2013). Furthermore, CsA immunoassay results have also been reported to be interfered by endogenous heterophile antibodies (Morelle et al., 2011). In the presented case, we took a stepwise approach to exclude other factors that may influence laboratory results and validating trough level reports with LC/MS/MS to reduce possibilities of misinterpretation (Netzel et al., 2014).

## Conclusion

In conclusion, this case report suggests probable drug interaction between etanercept and CsA that is mediated by cytokines. Etanercept initiation, resulting in resolution of inflammation, can lead to high clearance of CsA. Therefore, CsA blood levels should be closely monitored in patients who are receiving etanercept simultaneously or with active inflammatory diseases. Physicians and pharmacists should also be more vigilant in such patients as the co-administration of anti-cytokine therapies could significantly affect CsA concentrations and lead to supra- or sub- therapeutic levels. Additionally, in such cases, monitoring cytokine levels may be useful to detect potential drug-cytokine interaction.

## Data Availability Statement

All datasets generated for this study are included in the article/**Supplementary Material**.

## Ethics Statement

Ethical review and approval was not required for the study on human participants in accordance with the local legislation and institutional requirements. The patients/participants provided their written informed consent to participate in this study. Written informed consent was obtained from the individual(s) for the publication of any potentially identifiable images or data included in this article.

## Author Contributions

HW and DC drafted the manuscript and interpreted the data. JL performed analysis for sample determination. LL and CY have critically revised the article for important intellectual content. ZJ and BC provided technical support for sample determination. BZ provided material support in sample collection. All authors contributed to the article and approved the submitted version.

## Funding

This work was supported by National Natural Science Foundation of China (81873617), Guiding Medical Project of Shanghai Science and Technology Committee (18411961000) and Youth Science Fund of National Natural Science Foundation of China (81400687).

## Conflict of Interest

The authors declare that the research was conducted in the absence of any commercial or financial relationships that could be construed as a potential conflict of interest.
