# Supplementary material for: Probable Drug Interaction Between Etanercept and Cyclosporine Resulting in Clinically Unexpected Low Trough Concentrations: First Case Report
Source: Front Pharmacol. 2020 Jun 26;11:939. doi: 10.3389/fphar.2020.00939 (PMC7333231; doi:10.3389/fphar.2020.00939)
Supplement: Supplementary file 1 [file DataSheet_1.docx]

Supplementary Material

# Supplementary Data：

# LC/MS/MS determination of patient’s cyclosporine trough level: method validation and sample quantification

**Material and Methods**

*Chemicals*

CsA (130495-201303) was purchased from National Institutes for Food and Drug Control (Beijing, China). Ascomycin (20111117) as internal standard was purchased from Melonepharma company (Dalian, China). Methanol HPLC grade was produced by Tedia (Ohio, US). Blank blood from healthy donors was kindly supplied by Ruijin Hospital.

*Chromatograghic and MS conditons*

The MS instrument used was API4000 Qtrap Mass Spectrograph (Applied Biosystem, Toronto, Canada). The HPLC instrument used was UFLC Chromatographic System (Shimadzu Corporation, Japan), with two LC - 20 AD binary pumps, SIL-20 automatic sampler, CTO-20 column oven, CBM-20 Alite System Controller.

Chromatographic separation was performed at 40℃ using a column oven, on Eclipse XDB-C_18_ column 3.5μm，2.1 mm×100 mm (Agilent, La Jolla, USA). The mobile phase was composed by solvent A (HPLC grade water + 2mM ammonium formate) and solvent B (HPLC grade methanol). The mobile phase flow rate was maintained at 0.3 mL/min. The elution conditions were as follows: 0-0.5 min 20% B, 0.5-2.5 min 100% B, 5-5.6 min 20% B, 5.6-7min 20% B. Detector settings were ESI, positive polarity ionization; capillary voltage 3.5kV; desolvation temperature 500℃; GS1 pressure:40 psi; GS2 pressure:40 psi. CsA and ascomycin were detected in multiple reaction monitoring (MRM) mode using mass-to-charge (m/z) transition of 1219.4 → 1202.4 and 809.6 → 756.5, respectively. Collision energies were 53 eV and 29.9 eV， and declustering potential 30.7 eV, 97.7 eV. The scan dwell time was set at 0.2 s for every channel.

*Stock solutions, standards (STD) and quality controls (QC)*

Stock solutions of CsA and ascomycin (IS) were made in methanol, respectively, with a final concentration of 1.00 mg/mL and 4.10 mg/mL, and stored at −80℃ until use, no longer than 3 months.

Working solution of precipitant with IS was made with zinc sulfate solution (0.1M) and ascomycin (102.5 ng/ml) in HPLC grade water and then stored at 4℃ until use.

Serial dilutions of the stock calibrator in drug-free blood were done to obtain seven calibration standards (4, 10, 20, 40, 100, 400 and 1000 ng/mL). Separately, three QC samples (0.4, 4 and 40 ng/mL) were prepared as described for the preparation of the calibration standards. The solutions were stored at −80℃ until use, no longer than 3 months.

*Specificity and selectivity*

Interference from endogenous compounds was investigated by analysis of six different blood samples from patients without treatment of immunosuppressive drugs. The peak area response of interfering peak at the retention time of analyte should be less than 20% of the LLOQ for the analyte (CsA).

*Matrix effect*

The quantitative measure of matrix effect can be termed as matrix factor and defined as the ratio of the peak area response in the presence of matrix (measured by analyzing blank matrix spiked after extraction with analyte) to the peak area response in the absence of matrix (pure solution of analyte).

Matrix effect was investigated using five different blank blood. The peak areas of the standard solutions containing three different concentrations of a solution of water and methanol (50:50) and peak areas obtained from blanks post-extraction solution with the same amount of CsA were compared.

*Accuracy, precision, calibration and limit of quantification*

Intra-day and inter-day accuracy were determined by assaying five spiked blood samples at three different concentrations (QCs) for each drug. Accuracy was calculated as the percent deviation from the nominal concentration. Inter-day and intra-day precision were expressed as the standard deviation at each QC concentration. Each calibration curve was obtained using seven calibration points in duplicate. A quadratic regression was used for all curves in order to obtain the best fit for all calibration points.

The limit of detection (LOD) in blood was defined as the concentration that yields a signal-to-noise ratio of 3/1. The lowest concentration levels that could be determined with a percent deviation from the nominal concentration and relative standard deviation <20%, was considered the lowest limit of quantification (LLOQ).

*Recovery*

Recovery from blood, using the extraction procedures, was assessed by comparing the peak area obtained from multiple analyses of spiked samples (QCs) with the peak area from standard solution of all analytes in a solution of water and methanol (50:50) at the same concentration.

*Stability*

We have investigated stability on QCs for CsA within one month of storage at −80 ℃, assessed by variation of areas during validation period. The stability of CsA repeated freezing was also investigated. A post-extraction short stability (12 h) was also evaluated.

*Patients plasma samples*

Blood samples were collected in heparin tube and then stored at -80℃.

**Results**

Time of analytical run was chosen as 7 min, the analyte retention times were 4.77± 0.2 min for CsA and 4.56 min for ascomycin. Mean regression coefficient (*r*^2^) of calibration curves was higher than 0.99.

*3.1. Specificity and selectivity*

The tested six blank blood samples did not show any interference in the retention times analytes windows for each specified ion detected (Fig 1. A). Representative chromatograms of CsA and IS extracted of blank blood was shown in Fig 1. B. Representative chromatogram of CsA and IS extracted of the patient blood sample was shown in Fig 1. C.

*3.2. Accuracy, precision*

Results of the validation of the method are listed in Table 1 for CsA. All observed data (intra-day and inter-day precision) were all below 15.0%.

*3.3. Recovery and Matrix effect*

Multiple aliquots (n=5) at each of the three QCs amounts were assayed and mean recovery of three different concentrations of CsA were 89.45%, 85.50%, 91.97%. The matrix effect was 81.59%, 75.94% and 84.06% respectively, listed in Table 2.


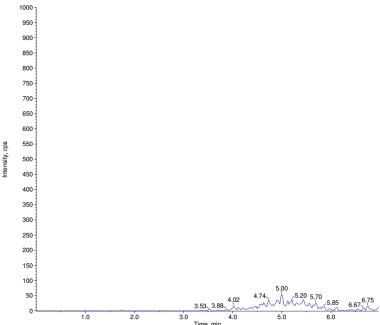

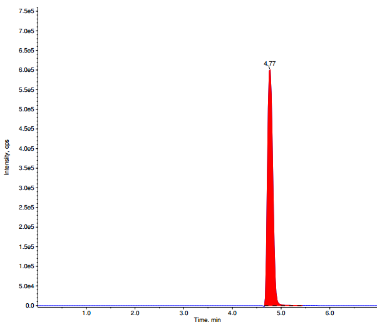

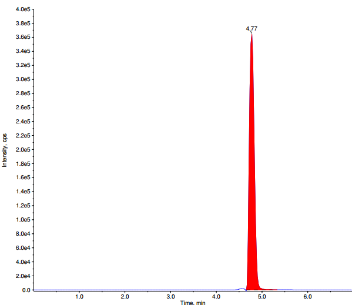


C

B

A

CsA


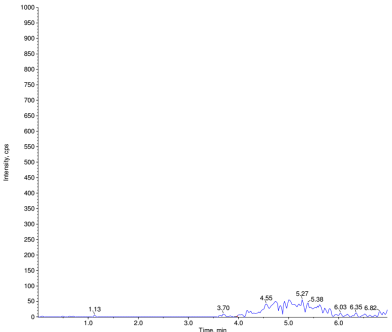

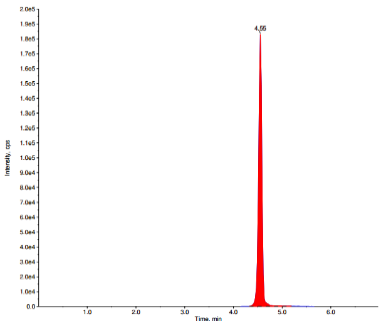

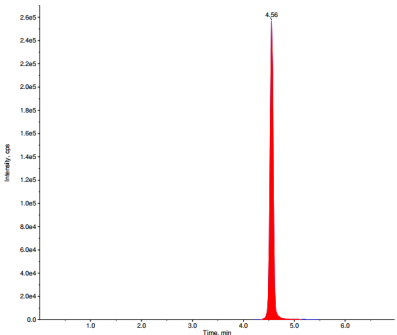


C

B

A

ascomycin

Fig 1. Chromatogram of cyclosporine (CsA) and ascomycin

(A) Blank blood sample;(B) Control blood sample;(C) The patient blood sample

*3.4 Stability*

No remarkable variation was observed for CsA from the analysis of stability on QCs within one month of storage at −80℃, repeated freezing and from the analysis of post-extraction short stability (12 h, autosampler) at room temperature (see Table 3).

Table 1. Results of accuracy and precision (n=5)

| Theoretical concentration  (ng·mL^-1^) | Intra-day assay | | | Day-to-day assay | | |
| --- | --- | --- | --- | --- | --- | --- |
|  | Concentration  (ng·mL^-1^) | Accuracy  (%) | RSD  (%) | Concentration  (ng·mL^-1^) | Accuracy  (%) | RSD  (%) |
| 8 | 8.50±0.65 | 106.23 | 7.71 | 8.29±0.95 | 103.66 | 11.48 |
| 80 | 81.32±3.10 | 101.65 | 3.81 | 87.58±6.42 | 109.48 | 7.33 |
| 800 | 844.75±14.03 | 105.59 | 1.66 | 878.43±36.25 | 109.80 | 4.13 |

Table 2. Matrix effect and extraction recovery (n=5)

| Concentration  (ng·mL^-1^) | Matrix effect  (%) | Recovery  (%) |
| --- | --- | --- |
| 8 | 81.59 | 89.45 |
| 80 | 75.94 | 85.50 |
| 800 | 84.06 | 91.97 |

Table 3. Results of stability study (n=5)

| Theoretical concentration  (ng·mL^-1^) | 12h at room temperature | | Repeated freezing | | One month at -80℃ | |
| --- | --- | --- | --- | --- | --- | --- |
|  | Concentration  (ng·mL^-1^) | Relative error  (%) | Concentration  (ng·mL^-1^) | Relative error  (%) | Concentration  (ng·mL^-1^) | Relative error  (%) |
| 8 | 7.71±0.36 | -3.60 | 8.31±0.22 | 3.85 | 7.34±0.21 | -8.30 |
| 80 | 87.22±2.06 | 9.03 | 86.42±2.43 | 8.03 | 71.42±1.47 | -10.73 |
| 800 | 847.20±27.04 | 5.90 | 900.6±20.06 | 12.58 | 766.25±8.54 | -4.22 |

3.5 Analysis of plasma sample

The blood concentration of CsA of provided patient’s sample was 59.4ng/mL.

**Reference**

Zhai XH, Liu XX, Lu JQ, *et al*. Establishment of LC-MS/MS assay for the determination of blood immunosuppressive agents level simultaneously. *Chin Hosp Pharm J*. 2019;39(8):774-780

# Supplementary Table

Table 1. Patient’s laboratory results of cytokine levels, inflammation indicators and hepatic/renal function.

| Days | Cytokines | | | | | | | Inflammation indicators | | Hepatic/renal function | | | |
| --- | --- | --- | --- | --- | --- | --- | --- | --- | --- | --- | --- | --- | --- |
|  | TNF-α | SIL-2R | IL-8 | IL-6 | VEGF | IL-2 | IFN-γ | CRP | ESR | ALT | AST | ALP | SCr |
| Day -224 | **11.6** | 580.0 | 19.6 | 2.9 | 130.3 | 142.8 | 12.0 | 3.2 | 17 | 38 | 23 | 83 | **110.7** |
| Day -126 | < 4.0 | 223.0 | 30.1 | 2.0 | 71.9 | **257.6** | 10.8 | 3.9 | 20 | 24 | 25 | 98 | **120.5** |
| Day -68 | **14.2** | 412.0 | 13.3 | 2.4 | 190.7 | **742.0** | 14.0 | 0.9 | 14 | 47.03 | 24.11 | 102.15 | **120.4** |
| Day 73 | **7.15** | 358.0 | 6.64 | 3.29 | 132.0 | **301.7** | 12.2 | <0.5 | 15 | 22 | 10 | 89 | **98** |
| Day 143 | **11.6** | 514.0 | 7.56 | 2.0 | 98.5 | 108.9 | 13.4 | 2.1 | 9 | 21 | 16 | 97 | **121** |
| Day 199 | **12.8** | 539.0 | 9.40 | **6.2** | 104.0 | **364.9** | 15.1 | **9.22** | 14 | 13 | 17 | 107 | **110** |
| Day 297 | 7.04 | 452.0 | 28.0 | **5.48** | 148.5 | **257.0** | 11.2 | 3.21 | 10 | 20.44 | 14.79 | 83.29 | **114.7** |

Note: 1.TNF-α stands for tumor necrosis factor α, SIL-2R stands for soluble interleukin 2 receptor, IL-8 stands for interleukin-8, IL-6 stands for interleukin-6, VEGF stands for vascular endothelial growth factor, IL-2 stands for interleukin-2, IFN-γ stands for interferon gamma. CRP stands for C-reactive protein, ESR erythrocyte sedimentation rate, ALT stands for alanine aminotransferase, AST stands for aspartate aminotransferase, ALP stands for alkaline phosphatase, SCr stands for serum creatinine.

2. Day zero set as first day with cyclosporine administration. Values outside of reference range are in **bold** characters.

3. Unit for cytokine levels is pg/ml, for CRP is mg/ml, for ESR is mm/h, for ALT/AST/ALP is U/L, for SCr is μmol/L. Reference range for each indicators are as following: TNF-α: < 8.1 pg/ml, SIL-2R: 223-710 pg/ml, IL-8: <62 pg/ml, IL-6: <3.4 pg/ml, VEGF: <200 pg/ml, IL-2 <200 pg/ml, IFN-r <20 pg/ml; CRP: 0-8 mg/ml, ESR: 0-21mm/h; ALT: 13-69 U/L, AST: 15-46, ALP: 38-126 U/L, SCr 57-97 μmol/L.

Table 2. Medication reconciliation and compliance assessment result of patient

| Medication | Dosage and duration | Compliance rate (%) |
| --- | --- | --- |
| etanercept | 25mg biw subq from July 2014 to July 2015  25mg qw subq from July 2015 to October 2017  25mg every 10 days subq from until now | 70% |
| cyclosporine | 75mg bid po since August 2018 until now | 100% |
| methylprednisolone | 32mg to 8mg (tapering down) qd po from August 2018 to November 2018 | 100% |
| metformin | 500mg qd po since 2016 | >95% |
| levoamlodipine | 2.5mg qd po since 2014 | >95% |
| losartan | 50mg qd po since 2015 | >95% |
| celecoxib | 200mg po qd, before July 2014 | discontinued |
| sulfasalazine | before July 2014, dosage not clear | discontinued |
| methotrexate | before July 2014, dosage not clear | discontinued |

Table2. CARE Checklist for the presented case report

| **Topic** | **Item** | **Checklist item description** | **Reported on Line** |
| --- | --- | --- | --- |
| **Title** | **1** | The diagnosis or intervention of primary focus followed by the words “case report” | **yes** |
| **Key Words** | **2** | 2 to 5 key words that identify diagnoses or interventions in this case report, including "case report" | **23** |
| **Abstract**  **(no references)** | **3a** | Introduction: What is unique about this case and what does it add to the scientific literature? | **28-31** |
|  | **3b** | Main symptoms and/or important clinical findings | **28-31** |
|  | **3c** | The main diagnoses, therapeutic interventions, and outcomes | **31-34** |
|  | **3d** | Conclusion—What is the main “take-away” lesson(s) from this case? | **36-39** |
| **Introduction** | **4** | One or two paragraphs summarizing why this case is unique (**may include references**) | **59-60** |
| **Patient Information** | **5a** | De-identified patient specific information | **yes** |
|  | **5b** | Primary concerns and symptoms of the patient | **63-64** |
|  | **5c** | Medical, family, and psycho-social history including relevant genetic information | **64-69** |
|  | **5d** | Relevant past interventions with outcomes | **64-69** |
| **Clinical Findings** | **6** | Describe significant physical examination (PE) and important clinical findings | **69-71** |
| **Timeline** | **7** | Historical and current information from this episode of care organized as a timeline | **figure 1** |
| **Diagnostic Assessment** | **8a** | Diagnostic testing (such as PE, laboratory testing, imaging, surveys) | **71** |
|  | **8b** | Diagnostic challenges (such as access to testing, financial, or cultural) | **not applicable** |
|  | **8c** | Diagnosis (including other diagnoses considered) | **not applicable** |
|  | **8d** | Prognosis (such as staging in oncology) where applicable | **not applicable** |
| **Therapeutic Intervention** | **9a** | Types of therapeutic intervention (such as pharmacologic, surgical, preventive, self-care) | **72-76** |
|  | **9b** | Administration of therapeutic intervention (such as dosage, strength, duration) | **72-76;100-110** |
|  | **9c** | Changes in therapeutic intervention (with rationale) | **74-76** |
| **Follow-up and Outcomes** | **10a** | Clinician and patient-assessed outcomes (if available) | **80-82;91-96** |
|  | **10b** | Important follow-up diagnostic and other test results | **114-126** |
|  | **10c** | Intervention adherence and tolerability (How was this assessed?) | **98-113** |
|  | **10d** | Adverse and unanticipated events | **74-75; 76-80; 107-111;** |
| **Discussion** | **11a** | A scientific discussion of the strengths AND limitations associated with this case report | **162-163;181-185;198-214;** |
|  | **11b** | Discussion of the relevant medical literature with references | **164-181;186-197** |
|  | **11c** | The scientific rationale for any conclusions (including assessment of possible causes) | **215-225;227-229** |
|  | **11d** | The primary “take-away” lessons of this case report (without references) in a one paragraph conclusion | **229-234** |
| **Patient Perspective** | **12** | The patient should share their perspective in one to two paragraphs on the treatment(s) they received | **108-113** |
| **Informed Consent** | **13** | Did the patient give informed consent? Please provide if requested. | **yes** |
